# Drinking Carling Out of Stella Glasses: People and Place in the Missing Middle

**DOI:** 10.3389/fsoc.2020.534515

**Published:** 2021-01-20

**Authors:** Kathryn McEwan

**Affiliations:** Department of Nursing, Midwifery and Health Northumbria University Newcastle, Newcastle upon Tyne, United Kingdom

**Keywords:** social change, social mobility, meritocracy, place, missing middle

## Abstract

As trends of social and economic change allow precarity to inch into the lives of those who may have been more accustomed to security (Standing, 2011, 2014), this paper addresses the response of some young people who are caught “betwixt and between” in potentially liminal states (Turner, 1967). Those whose families have undertaken intra- or intergenerational social mobility and who have made a home in a place, Ingleby Barwick in Teesside, that seems to be of them and for them—an in-between place that is seen as “not quite” middle or working class. This paper draws data from a research project that adopted a qualitative phenomenological approach to uncover the meaning of experiences for participants. Methods included focus groups and semi-structured interviews through which 70 local people contributed their thoughts, hopes, concerns, and stories about their lives now and what they aspire to for the future. Places, such as the large private housing estate in the Northeast of England on which this research was carried out, make up significant sections of the UK population, yet tend to be understudied populations, often missed by a sociological gaze attracted to extremes. It was anticipated that in Ingleby Barwick, where social mobility allows access to this relatively exclusive estate, notions of individualism and deservingness that underlie meritocratic ideology (Mendick et al., 2015; Littler, 2018) would be significant, a supposition borne out in the findings. “Making it” to Ingleby was, and continues to be, indicative to many of meritocratic success, making it “a moral place for moral people” (McEwan, 2019). Consequently, the threat then posed by economic precarity, of restricting access to the transitions and lifestyles that create the “distinction” (Bourdieu, 1984) required to denote fit to this place, is noted to be very real in a place ironically marked by many outside it as fundamentally unreal.

## Introduction

This paper presents and discusses the experiences of those who find themselves in the “missing middle” of class analysis and sociology. Those who “drink Carling out of Stella glasses,” as a participant shared, when attempting to describe a middling (or inauthentic) status of place and people. Drawing together concepts of the “missing middle,” meritocracy, and social mobility, which interact, overlay, and obfuscate class (dis)advantage in the “borderlands” between the traditional working and middle classes, this article presents analysis from an empirical research project asking the following questions: What is it like being in the missing middle? How secure is the status? What, ultimately, does this mean for people's understanding of fairness, choice, and advantage? Specifically, these questions are asked of a population in a place where “middling” dispositions appear to manifest largesse, seemingly built in to a private housing estate designed to be intentionally different from working-class neighborhoods.

Liminality is an anthropological concept that describes a “betwixt and between” status. Specifically, it denotes progressing through the in-between stage of a rite of passage, when one has ceased to be who/what one once was yet has not completed the full transition into who/what one will go on to be (Turner, 1967). Given this, liminality is not used in its strictest sense here (a temporary situation in which, should the progression halt, the person instead becomes “marginalized”), but rather, it is adopted to refer more generally to being “betwixt and between,” offering a temporal and spatial notion for explaining in-betweenness. That said, feelings of not quite belonging on either side for intragenerational social mobility and how this may relate to the marginalization of those who are unsuccessful in social mobility is interesting nonetheless and would warrant further investigation.

The concept of the “missing middle” used herein has two similar but discrete meanings, alluding to both sociological analysis and social class stratification. For the former, it refers to an in-between status, between working and middle class, in which people feel themselves to be not quite fully one or the other oftentimes because of the accumulation (or not) of resources or due to inter-/intragenerational mobility and associated class cultural legacy. For the latter, it refers to claims of an apparent lack of sociological analysis of those in the middle of UK society by a discipline attracted to extremes (Byrne, 2005). As class has returned to the forefront of sociological analysis, it is hoped this contribution adds to the continued growth of “middling” sociology.

Class location is perhaps most messy for those on the borders of “traditional” class categories. Social positioning blurs as people move over the working–middle class boundary, backward and forward, taking up characteristics of both sides. Consequently, this is what Tyler (2015) describes as Bourdieu's “classificatory struggles” most often on display, when people and groups work intensely to maintain the boundaries between “them” and “us.” For those in the working–middle class border territories (geographically and sociologically), the power, privilege, self, and identity resources available are muddled. Bradley and Waller (2018) note their use of the “muddle in the middle” category for their group of participants who were not easily placed in traditional middle- or working-class groupings. This missing middle, of status and investigation, are those who live in between and were often underrepresented in research and writing before the 2010s, yet the ordinary and unspectacular make up much of the UK population (Byrne, 2005; Roberts K., 2013; Roberts S., 2013).

This paper recognizes class as existing through “base” and “superstructure,” which shape and form one another (Williams, 1973). Further, as explained by McGuigan and Moran(2014, p. 175–178), drawing on William's “notion of culture that […] we must analyse ideas, values and cultural forms in the social conditions of their production and circulation” and his argument “against the logic of concrete spheres and to focus on constitutive human activities.” Multiplicity and interrelatedness are key concepts that underpin the analysis and the stance taken by the author throughout. Furthermore, the argument developed herein draws on Tyler (2008, 2013, 2015) assertions that we recognize ourselves as who we are and our “fit” for place through what we *are not* as much as what *we are* and that actions are undertaken to create this “distinction.” The working classes, Bourdieu (1984) states, provide a negative reference point from which the dominant middle classes can define their cultural practices. Similarly, those who attempt upward social mobility face multiple accusations of pretentiousness and “getting above themselves” with many idioms deployed by locals (in this research and elsewhere) about the upwardly mobile working class, including “10 bob snobs” and “all fur coat, no knickers” (Lawler, 1999; Skeggs, 2004).

Although Bourdieu (1984, 1990) can be accused of underplaying some of the fundamental economic principles that underlie class, his work, particularly in *Distinction*, allows for investigation of the lived experiences of class in this “middling” research field, in which significant economic resources were rarely discussed as inherited (aside from hopes to provide this for subsequent generations through mortgaged property eventually acquired as capital). Furthermore, although an external labor market outside of their control was noted by participants to exist, it would be equally “explained away” with various ideas of agency frequently elevated above this structural constraint. Bourdieu's (1984. p. 56) states, “Taste classifies, and it classifies the classifier.” In the research interviews, people spoke of class, value (Skeggs, 1997, 2004, 2011, 2016), and deservingness in multiple ways. As such, Bourdieu's classificatory concepts offer a strong tool for analysis.

The research project from which this paper draws data rests on the assumption that the representation of the place, Ingleby Barwick, and the people who live there are a co-creation of those on both the inside and outside (Ritzer, 2003; Hodkinson, 2005). Bourdieu (1997); Bourdieu's (1984) theories of practice were guiding theories; he devoted much of his work, Ritzer (2003) claims, to building a bridge between structuralism and constructivism, demonstrating that objective and subjective structures are different yet operate in a dialectic relationship, influencing one another. Identifying these dialectic relationships is pertinent in a culture that often asserts the dominance of agency over structural inequality. Demonstrable in many forms, it is recognizable most recently, as Gill and Orgad (2018) explain, in the growth of language of agency and responsibility, currently visible in the increasing rhetoric of “resilience” that establishes this psychological resource as a limitless classless reserve when it is, in fact, primarily accessible to those who have multiple cultural and economic sources.

With access to economic resources in the missing middle most often predicated on employment income (rather than capital), any disruptions to that income stream could attract serious consequences. Therefore, should economic precarity be returning in new form, as Standing (2011, 2014) suggests, it is assumed this population would feel the consequences, be it in a direct and immediate financial sense or through heightened anxiety that they are vulnerable and exposed to risk. In a time when neo-liberal meritocratic ideologies, which push public accountability onto private individuals (Byrne, 1997, 2005; Littler, 2018), appear to be dominant streams of thought, it was presumed that this may be an essential element of the stories participants tell to make sense of their lived experiences.

It is important to investigate these life experiences and judgments (for Bourdieu, 1984, “classifiers” through “tastes”) as ideas of social mobility, meritocracy, deservingness, and value that underwrite UK politics and society. An individual's value, Skeggs (1997, 2004) states, is demonstrably linked to class status, and although class may not often be directly spoken, it is, nonetheless, objectively apparent in both occupation and where someone is consequently placed in a hierarchical structure. Research, therefore, is required into the motivations and meanings of the stratifications of the population that make up the “missing middle.” Those who reside in the class boundary land “betwixt and between” the higher working and the lower middle classes, predominantly where upward and downward social mobility takes place. Research such as this allows for interrogation of the lived experiences of (re)production of social divisions and inequalities and, as such, sheds light on the tensions and motivations that underlie people's “choices.”

Pakulski and Walters (1996) famously declared the death of class in the late 1990s in what was oft-claimed then to be a post-modern world (Beck, 1992). Indeed, then Labor UK Prime Minister Blair (1999) subsequently declared the class war to be over. Based on an ideology of aspiration, New Labor rhetoric, informed by the work of sociologist Giddens (1991), focused on the “responsibilities” of citizens, reducing the responsibility of the state to correct against capitalism. Pikkety (2014) demonstrates that inequalities have grown vastly since this period. The ideology that championed property ownership for “all” and widened opportunities to allow “all” to attend university has neither undone class (dis)advantage nor dismantled boundaries. The propulsion of class analysis and debate back onto the public and academic agenda on which this paper builds is enthusiastically welcomed by Skeggs (2014) as allowing the opportunity to discuss the structural opportunities and restrictions that are brought to bear on lives and to challenge the language used to deny class (dis)advantage.

## Methodology

The qualitative research project data from which this paper draws was designed to take a phenomenological and inductive approach to recognize the meaning of experiences for participants. The original research proposal was to investigate young people's responses to what Standing (2011, 2014) claims to be changing socioeconomic times. It set out to do that through answering an earlier challenge from Byrne (2005), who asked about the lives of people, young and old, who lived in a suburban place on Teesside. A place such as this, Byrne (2005) states, appeared confused and confusing in class status, and furthermore, places such as this made up significant sections of the UK population yet appeared often to be missed by a sociological gaze attracted to extremes. Consequently, these challenges were drawn together and intergenerational interviews undertaken to investigate how those in the “missing middle” explained life transitions (housing, family, education, and employment) they had taken (or were still to take).

The Teesside Studies have a strong vestige of youth transition research within local marginalized and excluded communities (see Webster et al., 2004; MacDonald and Marsh, 2005; MacDonald et al., 2005; MacDonald and Shildrick, 2007; Shildrick and MacDonald, 2013). Understanding neighboring young people's lives through transition is, therefore, in keeping with this legacy. There is a longer legacy of sociological investigation in Teesside into both place and work. Lady Bell's *At the Works* (Bell, 1907) describes the poor conditions of early industrial workers and their families who lived at the local capital's beck and call in Middlesbrough in the late 1800s. The history of place, people, and work in Teesside weave together to shape a “biography of the place” (Warren and Garthwaite, 2014; Warren, 2017) that shapes the lived experiences of those who reside therein. Following this, place held a prominent position in the research conceptualization and analysis as an active agent, its “biography” recognized as influencing and shaping the lives and choices of the participants.

One of the most prominent debates in current youth research, be it from a transitional, cultural, or social generation approach, is the argument of continuity and change: Researchers are asking whether life is quantifiably and/or qualitatively different for young people today compared to previous generations. Furthermore, youth transitions researchers debate whether “precarity” is simply a return to difficulties experienced by earlier generations or whether the features of current precarity are so unique that young people's transitions are demonstrably different and disrupted (MacDonald, 2011). To account for this, the research was designed to interview generations within families as much as possible. Although young people are the primary focus of this research, parents were also invited to discuss their transitions to allow for consideration of potential parallels or differences.

Silverman (2014, p. 39) states that “the facts we find in ‘the field' never speak for themselves but are impregnated by our assumptions.” In a contested place, such as Ingleby, where the nature of its meaning is claimed to be manufactured by residents from those on the outside, the ontological and epistemological position taken must be clear. Subsequently, this research adopts a somewhat nuanced approach, taking a variant of a “realist” approach, perhaps most close to “subtle realism,” which would assert that “an external reality exists but is only known through the human mind and socially constructed meanings” (Ormston et al., 2014, p. 5). Further, Ormston et al. go on to point out that “when so-called inductive researchers generate and interpret their data, they cannot do this with a blank mind” (Ormston et al., 2014). Subsequently, the author does not claim the research to be an entirely inductive analysis: the author knows the research field, is from the wider area, and knew many of the nicknames and ideas of place long before this research was undertaken as well as holding the author's own feelings, ideas, and assumptions about social class in contemporary Britain.

Bourdieu's (1984) theory of habitus, how he manages the interplay of the structure and agency dichotomy, is a fundamental theoretical basis of this research, and it is woven into the fabric of the research design, practice, and analysis. Dean (2017, p. 30, emphasis in original) explains the mathematical-style equation that Bourdieu (1984, p. 95) himself used, albeit he states he was not overly keen on how it simplified the position: “(Habitus × Capital) + Field = Practice [t]o break it down, we can see as (Who we are × What we've got) + Where we are = What we do.” This is useful as a clear underlying premise of the research, and also, Dean (2017) relates this to reflexivity in research: “(Personal biography/position x Research skills/resources) + Site = Research practice.” Research, Dean (2017, p. 8) demonstrates, is similarly an interplay of a person's own biography, social position, social cultural and economic resources, and the research field: “Reflexivity is the way we analyse our positionality, the conditions of a given social situation […] it is an exploration of the researcher's own place within the many contexts, power structures, and identities and subjectivities of the viewpoint.” As noted, the author knew this place as a local and, for full disclosure and positionality, would “move up” there.

Much as the author could not lay claim to an entirely inductive approach due to positionality, it was agreed during the ethics process to not anonymize the place discussed within the research: “Ingleby Barwick” in Teesside in Northeast England. This decision was based on the understanding that the explanations of this place and its relation to its wider locality required to give meaning to the findings would have easily given it away. In which case, any attempts at (or claims to) anonymity would be misleading. That said, to protect participants, all were given pseudonyms and minor changes made to identifying characteristics to protect confidentiality.

Fieldwork took place between 2015 and 2017, starting with focus groups, which took place at a local FE college, a local sixth-form center, and a youth center in Ingleby. From these, the framing and approach of the semi-structured interviews were devised. To understand youth transitions, how, what, where, when, and why forms of questioning are required. Participants were asked to talk through major transition stages (school to FE/HE, employment, housing, familial), be that looking backward, discussing current events, or projecting forward. Each interview was between 45 and 75 min and was individual or in a small familial group. The “corrections” and questioning of participants by other participants in real time that arose in the group interviews were of huge interest and added a richness to the responses. Overall, some 70 people contributed to the research, recruited through gatekeepers, the researcher's local connections, engaging with residents in community spaces (real and virtual), and snowball sampling. Informal interactions and conversations were written up as field notes, and stakeholder interviews took place with community members such as estate agents, retail managers, and local politicians.

Interviews were recorded and transcribed verbatim before qualitative data was analyzed following thematic analysis, searching for patterns and “instances” (Teddlie and Tashakkori, 2009). The emerging themes indicate an uncertainty or insecurity that demonstrated aspects of what Turner (1967) might explain as liminality, an in-betweenness, in four discrete areas that ranged from macro to micro: precarity as a consequence of larger economic and societal forces, an unsure class position or status, an ambiguity of place, and the uncertainty that can arise when undergoing transition from youth to adulthood. Subthemes were then identified under these overarching themes, noting how these elements played out individually and wove together. The analysis drew a complex and layered picture of people with varying resources across the main themes. It is suggests that none can be entirely understood without the other, and all underpin the lives and experiences of the families interviewed.

## Context: the Importance of (This) Place

### Ingleby Barwick

Although it has existed as a settlement and township in various guises over thousands of years, Ingleby Barwick began its current incarnation in the late 1970s when development started on a large private housing estate on a disused Thornaby airfield. It has been continuously built on by numerous housing developers since that time, growing over several farms. Locals share with hubris and horror that Ingleby was once the largest private housing estate in Europe, apparently losing the title to a German estate. Although it was not possible to corroborate this, it is a local tale commonly (re)told as a matter of fact.

Ingleby is situated in Stockton-on-Tees Borough in the Northeast of England, an area that falls within “Teesside,” a legacy place name taken from a local authority district in the late 1960s and early 1970s that encompassed the industrial towns situated around the River Tees. The original development was based on the creation of multiple “villages” that would make up a larger whole. Six “villages” have been built consecutively, the most recent addition is The Rings, currently viewed as the most desirable location to live according to a local estate agent, who shared that clients most often would prefer a smaller property on The Rings over a larger property in an older village at the same price. Many others move within the estate itself, taking part (intentionally or not) in an internal social mobility process. The estate is increasingly self-contained and hosts six primary schools, two secondary schools, general practice surgeries, dentists, vets, supermarkets, takeaway outlets, hardware stores, newsagents, funeral directors, estate agents, hairdressers, beauticians, churches, pubs, a golf course, and both a public and a private gym (the first Duncan Bannatyne, a famous Scottish entrepreneur whose surname is now synonymous with a chain of gyms and hotels, opened back in 1997).

During the time of the fieldwork, most house sales in Ingleby Barwick were detached properties that sold for an average of £228,411. The overall average price for a house in Ingleby in 2016 was £205,673. Comparing this with neighboring areas, this is nearly double the average for predominantly working-class Thornaby, which stood at £114,241, yet is significantly cheaper than Yarm, a predominantly established middle-class area where the average was £241,013. The average wage in Stockton on Tees in 2015 was £25,200 (Manpower, 2016), significantly higher than other locales in Teesside, with Middlesbrough's average £19,600. Given these salaries, it would likely take an above-average salary for Teesside in a double-income household with a significant deposit to be able to purchase a property here.

Ingleby is described as a “*nice*” place, a “*clean*” place, and a “*quiet*” place; these are fundamentally important words that were repeatedly used by participants. Such assertions were often employed when attempting to demarcate this place from other local working-class areas, those that still appeared stained by their industrial heritage. Ingleby feels demonstrably post-industrial; none of the local industrial works on which Teesside grew rich are easily visible on the skyline as they often are in parts of neighboring towns. This is analogous with the white-collar nature of the employment of many of the residents. A further frequently used description is that Ingleby is a “*neat*” place, as in tidy, and it is indeed highly ordered with planning restrictions on front fences and networks of connecting paths and street layouts. This sense of orderliness, alongside notions of safety, which arose from such discipline of people and place, is demonstrably appealing to many residents.

## Findings and Discussion

Liminality is an anthropological concept, first introduced by Van Gennep (1961) but made most famous by Turner (1967). It refers to an in-between stage of a rite of passage, wherein initiates cease to be who they once were yet have not become who they will go on to be. Liminality is about transition, it is about change, and it is also about ambiguity. I suggest that the uncertainty of living through precarious economic times while in a liminal status position through being indeterminately classed in position and place in addition to living through a liminal stage of life, the youth phase, generates insecurity. Liminality is useful for recognizing the concept of the “missing middle” in class stratification and as such for understanding that life experiences do not fall neatly into order and disorder or people themselves into clear classifications (Thomassen, 2015). The concept of liminality, in which people and places are betwixt and between, offers a spatial and temporal way of thinking about boundaries and thresholds, how and where individuals are located within society, and how they recreate structure. Such an idea offers a useful tool for thinking about youth transitions alongside other transitional states, such as new spaces and places and time periods as well as experiences of social mobility. The argument presented here is that young people in Ingleby are subject to four levels of “liminality,” or transition, and that this impacts, interacts with, and informs their “choices” and sense of value and being.

These levels of liminality range from macro to micro in scale and can be experienced and understood objectively and subjectively. They are, first, the phenomenon of precarity; this is manifest on social and individual levels and is in regard to tangible change in notions of security and stability, a consequence of late capitalism. Second is class in the middle; for those in the higher stratifications of the working class and the lower stratifications of the middle class, there is much internal mobility alongside apparent confusion and hybridity in terms of status, identity, and access to economic resources. Third, place and its meaning and purpose is in active construction in a new estate that appears to offer itself as a post-industrial place for a post-industrial people. The final phase is the youth phase of life itself, in which young people undertake multiple transitions in education, employment, housing, and family. These levels of liminality are claimed herein to be inextricably linked, each crucial for understanding the impact of the other.

The following draws on data from all four themes, which became “levels,” to attempt to demonstrate the multiple ways that tensions across the levels pulled against each other or could be collectively mobilized to manage uncertainty. Place was often a leading level; it was found that the place Ingleby presented a means of creating distinction that was well-recognized inside and outside of the estate. Therefore, even those who distanced themselves from such cultural practices were nonetheless aware of them and their use. Importantly, although they may not have agreed on the meaning and extent, all participants felt conditions were different intergenerationally and principally agreed that younger people faced different structural (dis)advantages when making housing, education, and employment transitions. In discussion, these structural circumstances were often mediated through ideas of individualism and cultural practices (Bourdieu, 1984, 1990) tied to deservingness and difference. This paper builds on earlier work in which the author suggests these beliefs and practices are drawn together into the creation of a “moral place for moral people” (McEwan, 2019). The geography matters, and it is demonstrably and actively mobilized by insiders and outsiders for the mapping of social class and stratification (Wills, 2008).

## The (Un)Real

Participants in the interviews often appeared to avoid provoking forms of class shame and stigma (Jensen and Tyler, 2015) attracted from being perceived to be middle or working class by establishing a “middling” position. Ingram (2011), using Bourdieu's concept of habitus, describes a “third space” for the socially mobile, outside of the working- or middle-class habitus, which can be created through a “habitus tug.” Similarly, Abrahams and Ingram (2013, p. 145) explain that, by being forced into a new field, a person is made to consider not only “what is novel in that space,” but that it also “creates a new lens to look at where [they] have come from.” In this research, when explaining their class positioning in interviews, participants often drew together what they saw as the “best bits” of working- and middle-class characteristics to create this middling status, those that most closely allied with meritocratic ideas of choice and deservingness. This practice appeared to offer them a strong basis for positive conceptualization of self and for societal participation yet also attracted accusations of inauthenticity.

It was Brent (26) who perhaps most neatly brought together these ideas of the (un)real, drawing together what I would suggest as multiple layers of liminal in-betweenness of class, place, and people:

Kathryn: “*Do you think they [people from Yarm] look down on people in Ingleby?”*

Brent: “Probably yeah, the thing is though I can understand why, everyone is financed up to death, everyone has all these houses and flash cars, and they are all just drinking Carling^*^ *out of Stella glasses and stuff.”*

Kathryn*: “If you had to say what class people on Ingleby are what would you say?”*

Brent*: “If there was a class between lower and middle I would put them in there, cos I think a lot of people think they are middle class on Ingleby, but I would say they are in between, because the housing and that they probably are middle class, but their personalities, really friendly, I would associate that with lower class people. Like if it was a Venn diagram thing, I would put them in the middle of it.”*

^*^Carling and Stella Artois are both brands of lager sold in the UK. In a major supermarket in England at the time of writing 10 × 440 ml cans have only have a £1 difference in price (both under £10). Marketing is consistently different for these brands. Stella is advertised as a continental product and used the slogan “reassuring expensive” from 1982 until 2007, and Carling advertising is focused on football, pubs, and “banter.”

## Bit of Both

Rebekah's (22) parents, she shared, told her she was on the “*boundary”* of middle and upper, and this boundary concept was also referred to by Annie (52), who described her class as “*borderline working-middle class*.” Richard (55) used language that described his position in a physical manner; he might be “*leaning*” toward the middle class, he said, but he would not define himself as such, his feet seemingly rooted in their working-class preposition. Over his interview, Richard frequently used the history, geography, and class embodiment practices that Emery (2019) suggests as a useful mode of analysis for deindustrialization and class.

Such forms of intermediate positioning were often discussed as a consequence of familial amalgamation, so parents or grandparents of differing class status and backgrounds were blended to create an intermediate or liminal transitional position through that heritage mix. Warren (18) describes,

“*My stepdad has come from a very working-class family, my mum a more upper-middle-class family, and now she is a very happy medium almost” (Warren, 18)*.

Jonny (21) and Francesca (25), brother and sister, describe a similar circumstance although one that also includes the important reflection of what this means for them intergenerationally. When I asked them what they thought their class status was, they answered,

Francesca: “*How many different ones are we going for? As I wouldn't say we were quite middle class, but I wouldn't say we were working either. Probably lower middle class.”*

Jonny: “*I would say working class for me.”*

Francesca: “*It depends on what you say by working class though cos for me, some people have opinions of the working class as those you see every day that go into the benefits office. I have heard it called in the* Daily Mail *the non-working class. A lot of people have an opinion of them as being the working class, and I don't, wouldn't consider myself that, not that there is anything wrong with it, but I don't think we are quite middle class, like your Middleton type family people, we are not up there.”*

Jonny: “*I don't know because I go to work, I am on minimum wage and stuff, like I don't know, I don't know what the definition of a working-class person is, but I think I don't have my own house yet, I don't really have a lot.”*

Francesca: “*Alright, well take it as us as a family as a whole, so me, you, mum, Mike*.

Jonny: [unsure tone] “*working middle?”*

Francesca: “*Yeah, that is what I mean, so in-between, not quite middle class not quite working class, so I would put us in between.”*

Jonny and Francesca were both in working-class occupations, yet their parents were both senior managers with high salaries. Francesca believed that she and her brother would do well-over time, so their current occupations were not reflective of who and where they were in a bigger sense; she would “work her way up” as her parents did.

## Virtue

Social mobility and fluidity have been presented to the UK public as an unquestionable good, purported to offer individuals the opportunity to fulfill their potential, and as such, to “better” themselves (Bukodi et al., 2015; Friedman, 2016). Such ideas are determined in government policy through a non-departmental public body named since 2016 as the Social Mobility Commission, previously the Social Mobility and Child Poverty Commission in the period 2012–2016 and before that in the period 2010–2012 as the Child Poverty Commission. Allied to the meritocratic model, it is suggested that this ensures those who hold the top positions in society do so as they have the right skills, not just the right parents, and as such appears (on the surface at least) to be both socially just and economically efficient (Cabinet Office, 2009, 2012).

However, the social mobility project has been demonstrably unsuccessful on many counts. A 2017 Social Mobility Commission report, *Time for Change: An Assessment of Government Policies on Social Mobility 1997–2017*, found that, after 20 years of policies to attempt to support social mobility, young people's wages had fallen by 16% since 2008, sitting below 1997 levels in real terms; that more new apprenticeships had gone to older workers than younger; and that graduate outcomes for disadvantaged students had demonstrated only very minor improvement over the period. Indeed, by the time of the sixth State of the Nation report by the Social Mobility Commission (2019, p. 2), the foreword states it “lays bare the stark fact that social mobility has stagnated over the last 4 years at virtually all stages from birth to work.”

Whether it is objectively achievable or not, implicit in the idea of social mobility is a social hierarchy of value; it implies that those left behind, or who a person was before they undertook upward mobility, was deficient or less valuable (Lawler, 1999; Skeggs, 2004). The interview findings reflect these ideas; structural inequality is rarely referred to, and instead, most inequalities are individualized and/or pathologized. Although many held precarious class positionality and were in their current class locations through reliance on precarious resources, such as full-time stable employment income, mortgaged homes, or financed lifestyles that could easily be lost in an economic downturn, the sentiment behind many interviews was that the “deserving” could and would rise out of the lower stratum of the working class. Lipsey (2014, p. 37) suggests meritocracy, the ideological idiom of socially mobility, to be a toxic ideal: offering “equality of opportunity […] combined with gross inequality of outcome, is the worst possible recipe for a harmonious society.” However, social mobility was almost universally welcomed as a social good in the interviews, albeit as a personalized burden.

Meritocracy is a fundamental element of neo-liberal ideology, making a virtue of the valor of striving (Shildrick et al., 2012), which subsequently leads to deservedness (Walkerdine, 2003). Should an individual fail to succeed in such a belief system, guilt is placed squarely at the individual's own feet. It was anticipated that, in Ingleby, where social mobility allows access to this relatively exclusive estate, that the notions of individualism and deservingness that underlie meritocratic ideology would demonstrate themselves to be significant, and this supposition was borne out in the findings. “Making it” to Ingleby was and continues to be indicative to many who live there and those who know this place locally of meritocratic success. However, the anti-pretentiousness (Lawler, 1999) doctrine of the working class remains in tension with the desirability of mobility.

## Class Trickery or a Prize Worth Winning?

Brent (26) had been successful in his career to date; he had secured a well-resourced apprenticeship and had recently been appointed to a well-paid permanent contract on completion. Now in skilled employment, having been a call center operative previously, it would be fair to say he had undertaken upward social mobility, yet the thought of making this change was not attractive to him in the way it was to Jonny and Francesca, and he described it as an unwanted fit:

“*I don't want to be associated with them people though. I like the lower class, they are more friendly, aren't they? They don't look down their nose at ya; you always find that people with nothing are always a lot friendlier, they are always happy to talk to you, have a laugh with you. Even just total random people waiting in the queue or whatever, but I don't think you get that with middle-class people, they keep themselves to themselves in public” (Brent, 26)*.

Undertaking social mobility seemed to be only genuine to many participants when it was undertaken intergenerationally, not intragenerationally. Such movement allowed time for a new habitus to form for the new generation as Richard (55) appeared to describe when he said it allowed the “*roots to fade*.” Emery (2019, p.3) notes that class includes “temporal processes of history and memory as well as geographical lineages.” Richard's son Matt (22) seems to recognize these memory roots:

“*I think I consider myself more of a working class if it was just me, but with my parents, like the way they are, I think it's aiming more towards middle class if anything […] I know my granddad on my dad's side is working class*, [Cumbrian town] *has always been a working town and a working-class place. So, I would say before he moved here my Dad would have been working class, but he has bettered himself by coming here” (Matt, 22)*.

Conspicuous consumption in this middling place and people was frequently remarked on by participants in the interviews. Rebekah (22) discussed being in the middle as a potential opportunity, which allowed for slightly higher economic resources that could be converted for use. However, she believed this did not translate into passing through a liminal or intermediate position or phase into a new genuine position and so undertaking a *complete* form of social mobility:

“*A hundred years ago, class was just money [.] whereas you can sort of trick people now, you can have a really nice car, and you can have a really nice house and all of this” (Rebekah, 22)*.

This class masking was also noted by Melissa (28), who had recently moved to Ingleby from a traditional working-class area and who described uncovering this class “trickery,” where a middle-class habitus was being performed so apparently effortlessly it passed as “genuine” at first glance:

“*I spoke to a lady on the school playground, and I thought she was going to be a doctor or something and she said oh yeah I am a nail technician and I thought, what, I thought you were going to tell me you were a doctor, the way that some people act is that they put themselves a lot higher than other people” (Melissa, 28)*.

Alternatively, some do not even attempt this “mask.” Brent (26) shared those using economic capital alone could not “pass” as having undertaken complete social mobility as they have not utilized all the capitals (Burke, 2015) required:

Brent*: “I mean look at some of the offshore lads, they earn a fortune, but they are thick, they are common, they are horrible, aren't they?”*

This notion of incompleteness of identity and status, of the masquerade of one thing as another and the associated pitfalls, begs the question of whether class is indeed mutable (Byrne, 2005) and whether upward social mobility is a feasible or attractive goal for any individual. Symbolic capital is the total of Bourdieu's (1984); Bourdieu (1990) economic, social, and cultural capitals converted to power, but it requires external validation. Skeggs (2015) states that such power can ascribe value through its sights, shapes, and sounds; furthermore, she later explains (2016, p. 5) that value can be “structured by use (what we do) and exchange (the value that can be realized).” Therefore, an upwardly mobile population, such as Ingleby's, would not possess the full symbolic power to legitimate its value and undertake complete class transitions.

Skeggs (2016, p. 4) ties the establishment of the middle class to the 1832 Reform Act, which gave them political representation through private property ownership and subsequent access to “the symbolic means for legitimation.” Middle-class personhood requires multiple resources as well as individualism, governmentality, and the ability to command a moral authority. Furthermore, the middle-class protects its interests in this personhood through “processes of exclusion and legitimation, and through symbolic boundary marking” that delegitimates the working class (Skeggs, 2016, p.11). This produces a class divide that most on Ingleby would find impossible to traverse entirely and so plays an active part in the creation of a “betwixt and between” class position and stratification for those who have enough resources and power to leave much working-class exploitation and stigma behind them yet cannot fully enter the middle class.

## Just Holding on

For all those who indicated this liminal and indeterminate class stratification or position as incomplete or some form of “class trick” or masquerade, the societal and familial pressure to undertake upward social mobility did not appear to be abated. Rebekah (22) explained she felt under pressure from her parents to continue, or at the least sustain, their attempts toward upward social mobility. Furthermore, as her grandmother resides in a far “less than” place, she feels this as a spur, reminding her that, if she does not work hard, she may have to face downward mobility and return to the original position of her father. In Ingleby, the geographical and economic proximity of those in a lower social class or even closer still in a lower stratification of the same class instigates what Ehrenreich (1989) characterized as a “fear of falling.”

Class position is a mark of success and is felt to be mutable to Rebekah's family; they expect their daughter to do better again, she explained. Her parents hope for her to be “*upper class*” through maintaining the trajectory they started. Rebekah notes that most overwhelming is how much of the responsibility for this rested on her own shoulders; further social mobility is a project of herself by herself. This is all indicative of what Byrne (1997) calls a weak version of social exclusion, transferring public accountability onto private individuals. Moving up the class hierarchy and so completing successful upward social mobility appears to be seen by Rebekah's family as not only still possible, but that any failure to do so would be a failure in her and by her (McEwan, 2019).

At the same time, inequality, as experienced by differing access to economic resources, has an inevitable stranglehold on many attempts by young people to undertake their own upward social mobility or indeed to simply avoid downward mobility. Phoebe's (21) father held a middle-class well-remunerated position, but he had left the family home a year previously and had ceased providing any financial support to his family. Phoebe's mother, in a working-class occupation, was unable to sustain the family's position on her own, which had an inevitable impact on what financial resources were available for their daughter to compete effectively in the art world in which she had chosen to train. This precarity did not seem to be particularly unique to this family. Indeed, one participant explained that the 2008 financial crisis had triggered what felt like a “flag day” on the estate with many houses (which often rely on dual-income households) going up for sale subsequently as the fallout and austerity that followed restricted employment opportunities and wages.

Phoebe described cut-price folders to display her work and the problems with traveling to university on unreliable and expensive public transport. She recognized that this puts her at a disadvantage among her middle-class peers, yet still spoke as if this was within her control to correct: “*But as long as I work hard, I will get there; it doesn't bother me.”* Although this may well be true in part, all the same, Phoebe frequently indicated individual and personal responsibility for the structural constraints she faces. This reflects the findings of Mendick et al. (2015) in their work on “aspirations,” where the right mindset and “hard work” were seen to be able to overcome all and any obstacles regardless of evidence to the contrary being all around. Similarly, it's a critical element for Gill and Orgad's (2018) “bounce-backable” woman.

The only young person who was comfortable talking about historic structural constraints was Jay (27), who had experienced upward mobility to Ingleby before returning to a local working-class area at the end of a relationship. Jay's conceptualization of class reproduction accessed through parenting advantage was expressed as he recalled outcomes of his school friends:

“*The kids whose parents had proper jobs, kids whose […] dads done real stuff, rather than just a job, like they had careers, kids whose parents had careers, they've all got careers now” (Jay, 27)*.

## “Social Magic”

It would appear that, although social mobility is accepted as an unquestionable good, in practice, this is rather about successful collation of resources that places people into an exalted stratification of the working class rather than moving people up into the middle class. Indeed, even if the resources had been available to manage the latter, which was unlikely, it was also resisted due to the stigma of anti-pretentiousness (Skeggs, 1997; Lawler, 1999). Even those who had undertaken the “hard work” and “aspired,” earning a higher position, as Mendick et al. (2015) identified was expected of them were castigated as undertaking forms of class trickery. As with Melissa's nail technician, it is a rarity that the socially mobile can affect what Lawler (2013), employing a Bourdieusian concept, names “social magic,” a naturalization of socially learned competencies, tastes, and dispositions of another class identity. Many are “caught out” in this untruth and reproached for attempts to cast themselves as not who they really are.

Ingram and Allen (2019, p. 729) use Lawler's (2013) social magic to explain how the hiring processes of some graduate recruiters conceal a desire for classed characteristics through transforming them “into ‘objective' criteria, which naturalize privilege as earned and developed skills.” These include “self-starter” and “entrepreneurialism,” individualized traits that rarely account for the economic capitals required to underpin them. Notions of meritocracy and associated ideology made themselves apparent through all the interviews with differing levels of clarity. A successful neo-liberal subject, Verdouw (2016) describes, takes on life as a project of self; they are almost wholly independent (outside of the family unit) and are personally responsible for their “choices,” as such, they are dependent on agentic action to ensure success. Any (lack of) success in this conceptualization is entirely personal, no matter where one starts or the resources they have at hand. These ideas arose in Elizabeth and her son Joe's interviews:

“*Say if there is someone who lives in Pallister Park [working-class estate in Middlesbrough] who wants the same, to achieve the same things, as someone who lived in Wynyard [exclusive housing estate near Sedgefield], if they both want it, I don't see why they couldn't” (Joe, 25)*“*We had nothing, [spouse] and I. We didn't have great upbringings, we've done alright, it was just hard work, no handouts” (Elizabeth, 56)*.

Josh recognized things might be more difficult but saw these as hurdles rather than barriers:

“*if someone is from a lower background or a higher background, I think you can get the same goals, it might be harder, but I think if you persevere with what you are doing I do believe that anyone from anywhere can get what they want” (Josh, 22)*.

This belief, that the power of aspiration and intention can (and should) overcome all, ignores what Hanley (2016) describes as the quite rational judgments and behaviors of those working-class young people who do not “aspire” to “higher” levels of education and occupation. Many working-class young people are keenly aware of the low expectations of them from larger society and so act accordingly. Harrison and Waller (2018) explain that young people do indeed aspire, but they also grasp what is objectively available to them and so temper those aspirations accordingly. Similarly, Papafilippou and Bathmaker (2018) demonstrate the accrual of additional capital alongside the academic qualification required for successful education to employment transitions is often made possible through creation of “a strong possible career self.” Built from the social psychology concept of Markus and Nurius (1986), this recognizes the concept of self as having an impact on future behavior given its grounding in a consistent self-belief in entitlement. The tension in expectations and possibilities in the “missing middle” could be acute, exposed to macro and micro precarity, and differing levels of resources.

## Stuck in the Middle

Although in an objectively relatively advantaged social group, Rebekah (22) explained her family had significant housing costs, which meant that, although they appeared “better off,” the family had little disposable income. She was aware that many in her peer group at university received more through the student loan system as they came from lower income households and, alongside living with meritocratic ideas of individualism, she described being lost in the middle managing this:

“*I do feel like the middle amount of people are getting a bit disadvantaged, especially in the North East cos is you are relying on your parents, and they just don't have that money, then you are kind of stuck, and you can't get on and yet everyone is saying you need to get on, it is you that needs to do it, so you kind of feel a bit stuck” (Rebekah, 22)*.

The objective circumstances of young people's lives in the “missing middle” means they have access to finite resources with which to enact (or maintain) social mobility and so be able to achieve or sustain “middling” class positions. Furthermore, this was frequently “explained away” by notions of positive individualism, “resilience” (Gill and Orgad, 2018), and the valor of striving (Shildrick et al., 2012)—all of which were reminiscent of the language or meritocracy and neo-liberal ideology that obfuscating class (dis)advantage.

Macro conditions, including a poor labor market, high house prices, and high personal debt, are in a dialectical relationship with micro conditions enacted through individualized responsibility for public issues (Byrne, 1997), which include subjective experiences of insecurity and uncertainty. Together, these left participants feeling stuck and, with a strong meritocratic ideology instilled in their understandings of participation and success, ready to attach blame to themselves or other individuals rather than structural (dis)advantage for any success or failure.

## Conclusion

This paper drew on data from a research project to consider how people explained and understood their lived experience of inter-/intragenerational social mobility in the “missing middle” of class and sociology. The data was collected between 2015 and 2017 as part of a qualitative phenomenological research project and included interviews of young people (up to 30) and their parents whenever possible to compare and consider differences. The field of study was a large private housing estate in Teesside called Ingleby Barwick with multiple schools and amenities, situated in between more traditional working- and middle-class areas in the region. The estate was known locally as a destination of social mobility, a place where one had “made it” by moving up and moving in.

The “missing middle” was described as a “betwixt and between” status of class—an often self-selected stratification, which participants describe as taking on a hybrid identity because of inter-/intragenerational mobility, which includes elements of both working- and middle-class cultural practices and practicalities. Another mode of the “missing middle” is the lack of sociological analysis in the middle of society by discipline attracted to extremes. The argument is developed that the “missing middle” is, therefore, an interesting site to research people's experiences in what many researchers, theorists, and commentators have claimed to be changing or at least challenging times.

This paper asks, what is it like being in the missing middle? How secure is the status? What does this mean for people's understanding of fairness, choice, and advantage? It answered that “middling” positions were purposefully sought out and claimed to attempt to gain control of an insecure and uncertain status and avoid class stigma and shame. Further, that this “middle ground” allowed for the denial of class (dis)advantage that was at odds with the meritocratic language of the neo-liberal ideology that were cloaked as “common sense.” Participants mobilized varying degrees of economic, social, and cultural resources across the “levels” of liminality to gain or maintain their position, yet revenue streams were not guaranteed, and the prospect of another “flag day” was a consistent specter on the horizon.

Much felt uncertain with the in-betweenness often felt to be rejected by those in the classes above and below them as unreal or inauthentic. Ingleby manifested largesse that one could “move on” and “move up” away from industrial working-class origins. It offered, from conception, a “neat” and “clean” estate, perhaps where you could keep your white collar immaculate unlike in the neighboring town known colloquially as “smoggy land.” Ingleby offered a place free of the stains of industrial heritage writ large on many working-class neighborhoods. However, the social mobility offered through meritocracy worked as an apologist and cover for social class, which, for all its denial of import, was nonetheless apparent in macro and micro experiences of lives.

The “levels” of liminality accorded participants with varying degrees of resources to mobilize. Participants often appeared to be involved in “classificatory struggles,” which provoked heavy defense of positioning and privilege in this class borderland. The residents who participated in the research discussed what cultural practices they recognized as being of the “missing middle” and what these meant to them (and outsiders). Overall, these created a “moral place for moral people,” that was aspirational in its presentation and participants. However, this was often described to be not genuine by residents, in relation to themselves, others, and how they were perceived by those on the outside. Those who were accused of “drinking Carling out of Stella glasses” were reproached as a pretend (or pretentious) group of people.

Given this, it is fair to ask, what is the win, and is it worth it? Insecure status was reflected in the wider economic circumstances, homes, and lives were often built on unstable resources, such as mortgages and wages. Opportunities for the younger generation to perpetuate or even, in some circumstances, maintain the social mobility started by their (grand)parents were contracting; young people's wages are lower in real terms than in 1997, and housing and services costs are high. However, such structural constraint was rarely discussed, particularly so by younger participants who instead often subscribed to neo-liberal notions of meritocracy that push public accountability onto the shoulders of private individuals. Class was thrown into a “middling” place as it was, all at once, agreed to be important and not important at all, claiming the liminal, in-betweenness allowed for the denial of class (dis)advantage, which was not in keeping with people and place.

For all this appears an unflattering picture of place and people; it's fair to ask what is so wrong with the attempts being made to secure any form of advantage of oneself or family? Rather, the research suggests the shame should not be on those who wish to accrue advantage, security, and avoidance of “smog” for them and their children, but a societal structure and governance that allows the acute risk of maintaining it to be so privatized. Meritocracy appeared to offer a sense of control of one's fate with inequality or injustice (and alternatively success) pathologized or individualized. In this belief system, the virtue of unquestionably striving would be rewarded with success, no matter that social mobility has, by most measures, stagnated.

“Making it” to Ingleby was (and continues to be) indicative to many who live there and those who know this place locally of deservingness. This matters as the missing middle make up significant numbers of the UK population, and understanding their beliefs and motivations is critical for social policy makers, academics, and those who hope to influence them to participate in politics with a potential new “flag day” on the horizon following the Covid-19 pandemic.

## Data Availability Statement

The raw data supporting the conclusions of this article will be made available by the authors, without undue reservation, to any qualified researcher.

## Ethics Statement

The studies involving human participants were reviewed and approved by Teesside University. Written informed consent to participate in this study and for publication of their verbatim quotes was provided by the participants.

## Author Contributions

KM undertook proposal, data collection, analysis, and wrote up of the empirical research this article is based on.

## Conflict of Interest

The author declares that the research was conducted in the absence of any commercial or financial relationships that could be construed as a potential conflict of interest.
